# E-/P-selectins and colon carcinoma metastasis: first *in vivo* evidence for their crucial role in a clinically relevant model of spontaneous metastasis formation in the lung

**DOI:** 10.1038/sj.bjc.6605492

**Published:** 2009-12-15

**Authors:** S Köhler, S Ullrich, U Richter, U Schumacher

**Affiliations:** 1 Institute for Anatomy II: Experimental Morphology, University Cancer Center Hamburg, University Medical Center Hamburg Eppendorf, Martinistrasse 52, 20246 Hamburg, Germany

**Keywords:** cell flow experiments, E-selectin, metastasis, P-selectin, tumour cell glycoconjugates, xenograft model

## Abstract

**Background:**

Interactions of endothelial selectins with tumour cell glycoconjugates have been shown to have a major role in tumour cell dissemination in previous experiments. However, experiments validating this observation were limited in value, as ‘metastases’ in these experiments were artificially induced by i.v. injection rather than developed spontaneously as in true metastases.

**Methods:**

Endothelial (E) and platelet (P)-selectin-deficient severe combined immunodeficient (scid) mice were generated and human HT 29 colon cancer cells were subcutaneously inoculated in these mice and in wild-type scid mice. Tumour growth, spontaneous metastasis formation in the lung and adherence of HT29 cells to E- and P-selectin under flow were determined.

**Results:**

The number of metastases decreased by 84% in E- and P-selectin-deficient scid mice, compared with wild-type scid mice. The remaining 16% metastases in the E- and P-selectin-deficient scid mice grew within the pulmonary artery and not in the alveolar septae as they did in wild-type scid mice. Flow experiments indicate that tumour cells roll and tether on an E- and P-selectin matrix similar to leukocytes; however, firm adhesion is mainly mediated in E-selectin.

**Conclusion:**

Our results indicate that E- and P-selectins have a crucial role in spontaneous metastasis formation. As the human HT 29 colon cancer cells are positive for the lectin *Helix pomatia agglutinin* (HPA), which identified the metastatic phenotype in earlier clinical studies, these results are of particular clinical relevance.

The prognosis of patients suffering from colon cancer with formation of widespread metastases is still unacceptably low (Chambers *et al*, 2002; Poston *et al*, 2008). A hallmark of malignant progression is the occurrence of altered glycosylation on the tumour cell surface (Altevogt *et al*, 1983; Konno *et al*, 2002). This altered glycosylation pattern of the tumour cells has been detected using lectins, the carbohydrate-binding proteins. The lectin isolated from the Roman snail, *Helix pomatia agglutinin* (HPA), has been particularly useful, binding preferentially to tumour cells of those patients with a poor prognosis caused by a wide metastatic spread of their primary tumours (Mitchell and Schumacher, 1999; Brooks, 2000; Konno *et al*, 2002). The association between HPA binding and metastasis observed *in vivo* also holds true in a xenograft animal model of human breast and colon cancer metastasis. In general, only those human breast and colon cancer cell lines metastasised in severe combined immunodeficient (scid) mice that were HPA positive (Schumacher and Adam, 1997). For further analysis of the metastatic process, we focused in this study on the metastatic and HPA-positive human colon carcinoma cell line, HT 29.

The reason why HPA binding to tumour cells is associated with their metastatic potential has remained unclear so far. HPA-binding sites are located at the tumour cell surface (Mitchell *et al*, 1995), and therefore may be involved in adhesive interactions of malignant cells with their environment. Blocking experiments with HPA showed that adhesion of breast cancer cells with HPA-positive glycotopes to TNF*α*-activated endothelial cells could be blocked by HPA, whereas this was not the case with HPA-negative breast cancer cells (Valentiner *et al*, 2005). As TNF*α* stimulates the expression of the endothelial (E) and platelet (P) selectins on the luminal surface of endothelial cells (Rajan *et al*, 2008), it is attractive to hypothesize that HPA-positive glycoconjugates exert an effect as ligands for the two selectins. These adhesion molecules bind to glycotopes on leukocytes and are known to be key players in endothelial cell interaction and in trafficking of leukocytes and in mediating tethering, rolling and endothelial activation (Sperandio, 2006). The aim of this study is to analyse the role of the selectins in the metastatic process of HT 29 colon cancer. Although some researchers found E-, P- and L-selectins to be crucial for metastasis formation in colon carcinoma (Napier *et al*, 2007), one recent study emphasises the role of E-selectin in the diapedesis of colon cancer cells through the vascular endothelium (Tremblay *et al*, 2008). However, the role of E- and P-selectin in colon cancer metastasis has not been analysed in a clinically relevant spontaneous metastasis xenograft model. We therefore developed an E- and P-selectin-deficient scid mouse strain, in which we could quantify the influence of those selectins on the formation of spontaneous lung metastasis of HT 29 tumour cells. As the functional role of HPA-binding glycotopes present on the tumour cells is the focus of this study, we focused on E- and P-selectin present on the host endothelia serving as possible binding partners for HPA-positive cancer cells. Although recent experimental data indicate that lymphocyte L-selectin–tumour cell interaction is of importance in colon metastasis formation as well (Resto *et al*, 2008; Thomas *et al*, 2008, 2009), it was not the subject of this study as homozygous scid mice contain few if any lymphocytes (Bosma *et al*, 1983), implying that L-selectin expression present on lymphocytes is not an immediate concern in our xenograft model.

## Materials and methods

### Establishment of the E- and P-selectin-deficient scid mouse strain

E- and P-selectin knockout mice (Jackson Laboratory, Bar Harbor, ME, USA, stock 002916) were crossbred with Balb/c scid mice from an in-house breeding colony. Their offspring until F5 were crossbred again and genotyped for E- and P-selectin-null mutants and for scid.

### Analysis of the E- and P-selectin-null alleles

Mouse DNA was extracted from tail biopsies of each individual after digestion in lysis buffer using standard phenol/chloroform extraction. Intact or null alleles of E- and P-selectin in scid mice were evaluated using PCR. Primers specific for the wild-type and mutant E- and P-selectin alleles, as described earlier (Frenette *et al*, 1996; Kim *et al*, 1998), were obtained from MWG-Biotech AG (Ebersberg, Germany). PCR reactions for amplification of P-selectin alleles were performed in 25 *μ*l sterile polymer PCR tubes containing 12.23 *μ*l distilled water, 2.5 *μ*l reaction buffer S ( × 10 conc.), 5.0 *μ*l enhancer solution P ( × 5 conc.), 0.52 *μ*l primer 1 (24 *μ*M), 1.47 *μ*l primer 2 (17 *μ*M), 0.6 *μ*l primer 3 (21 *μ*M), 0.5 *μ*l d-NTPs (10 mM), 0.16 *μ*l Taq DNA polymerase (5 U *μ*l^–1^) and 2.0 *μ*l of DNA extraction sample. Reaction buffer S, enhancer solution P, d-NTPs and Taq DNA polymerase were obtained from peqLab Biotechnologie GmbH (Erlangen, Germany; no. 01-1010, SAWADY Taq-DNA-Polymerase-Kit). PCR reactions for amplification of E-selectin alleles were performed similarly, except for 12.26 *μ*l distilled water, 0.6 *μ*l primer 3 (21 *μ*M), 1.0 *μ*l primer 4 (19 *μ*M), 1.32 *μ*l primer 5 (19 *μ*M) and 0.165 *μ*l Taq DNA polymerase (5 U *μ*l^–1^).

PCR was performed in the following steps:


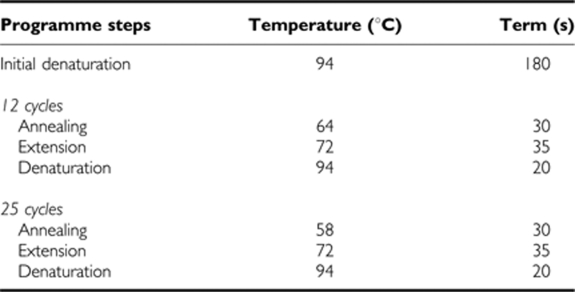


The PCR-amplified DNA fragments were analysed by electrophoresis in 1.5% agarose gel stained with ethidium bromide.

### Analysis of severe combined immune deficiency

Severe combined immune deficiency was verified by quantification of the level of mouse immunoglobulins in mouse serum using a dot blot assay as described earlier (Brown *et al*, 1997).

### Culture and inoculation of HT 29 cells

The human colon adenocarcinoma cell line HT 29 was purchased from the European Cell Culture Collection (Porton Down, Wiltshire, UK). Cells were cultivated, embedded in paraffin wax and, for inoculation, subcutaneously injected between the scapulae as previously described (Schumacher *et al*, 1994) in 6- to 10-week-old E- and P-selectin-deficient scid mice (*n*=30) and also in 29 scid mice of similar age as controls. Mice were provided with sterile water and food *ad libitum*. All manipulations were carried out aseptically and in accordance with the approval of the local animal welfare committee.

### Histology

Primary tumour, lung, liver, spleen and bone marrow of killed animals were removed, fixed in 4% formalin and embedded routinely in paraffin wax. For immunohistochemistry and standard H&E staining, 5 *μ*m thick sections were cut. The number of lung metastases was evaluated as previously described (Jojovic and Schumacher, 2000).

### Lectin histochemistry

Lectin histochemistry on paraffin wax sections was carried out as previously described (Brooks *et al*, 1996). For visualisation of the HPA-binding sites, the sections were treated with either an avidin horseradish peroxidase complex (Vector ABC kit; Vector Laboratories, Burlingame, CA, USA) followed by an incubation with DAB hydrogen peroxide for 10 min and a counterstaining with haematoxylin, or alternatively, an avidin alkaline phosphate complex using Naphtol-AS-bisphosphate and Mayer's haemalum as counterstain was applied. Hexatozised new fuchsin was used for simultaneous coupling. Slides were examined with a Zeiss Axiophot photomicroscope and documented digitally with the software axio.vision4 (Jena, Germany). The intensity of positive staining from no staining (0), weak (+) to very intense (+++) staining was recorded.

### Selectin immunoglobulin fusion protein histochemistry

Sections were deparaffinised and washed with lectin buffer (for details, see Brooks *et al*, 1996). Endogenous alkaline phosphatase (AP) was blocked with 20% acetic acid in 150 ml methanol for 3 min at room temperature (Brittan *et al*, 2002). After one further wash in lectin buffer, the sections were incubated for 30 min at room temperature with rabbit normal serum (no. X0902, Dako, Glostrup, Denmark), diluted 1 : 10 in Dako's antibody solution (no. S3022). The sections were then incubated for 1 h at room temperature or at 4 °C overnight with 4 *μ*g/ml^–1^ mouse P-selectin IgG fusion protein (no. 555294, BD Pharmingen, Franklin Lakes, NJ, USA) or 20 *μ*g/ml^–1^ mouse or human E-selectin/Fc Chimera (no. 724-ES, R&D Systems, Minneapolis, MN, USA), respectively. The sections were washed with TBS, followed by an incubation for 1 h at room temperature with a biotinylated rabbit IgG antibody against mouse (no. Z0259, Dako) diluted 1 : 200 in Dako's antibody solution. After one further wash in TBS, the sections were incubated for 30 min with an avidin-alkaline phosphate complex as described above. For the staining of cell cultures in chamber slides, the medium was removed by suction and the cells were then fixed for 10 min in methanol. After washing the chamber slides in PBS and in lectin buffer, further treatment was performed as described above.

### Double labelling with HPA and selectin fusion protein

Sections were deparaffinised, washed with distilled water and incubated for 15 min at 37 °C in lectin buffer with 0.06% protease XXIV (no. P8038, Sigma, St Louis, MO, USA) being added. After a wash under tap running water and another with lectin buffer, sections were incubated for 1 h with HPA and further treated with a biotinylated avidin-alkaline phosphate complex as described above. Enzyme reactivity of the latter was visualised by incubation for 15 min at room temperature with Fast Blue (no. 44670, Fluka Chemika AG, Buchs, Switzerland). The sections were washed under tap running water and with TBS, followed by incubation for 30 min at room temperature with rabbit normal serum (no. X0902, Dako), diluted 1 : 300 in Dako's antibody solution (no. S3022, Dako). Further treatment with the selectin IgG fusion protein and the biotinylated rabbit IgG antibody against mouse was performed overnight as described above.

### Labelling with anti-sLe^a^ and anti-sLe^x^ antibodies

Sections were deparaffinised, washed with TBS and incubated for 30 min at room temperature with rabbit normal serum and at 4 °C overnight with a primary monoclonal mouse antibody against human sialyl Lewis x (sLe^x^=CD15s, clone CSLEX1) (no. 551344, BD Pharmingen) or a monoclonal mouse antibody raised against human sialyl Lewis a (sLe^a^, clone 121 SLE) (no. ab3982, Abcam, Cambridge, UK), respectively, both diluted 1 : 100 in DAKO's antibody diluent solution. Further treatment was performed as described above.

### Analysis of circulating tumour cells

#### DNA extraction from murine blood and cultured human tumour cells

Representative blood samples were withdrawn from mice by puncturing the heart after general anaesthesia with ether. Approximately 1 ml of blood could be collected from a 25 g mouse. A total of 200 *μ*l of each sample was prepared for DNA extraction using the High Pure PCR Template Preparation Kit (no. 1 796 828, Roche Diagnostics, Penzberg, Germany). To establish a standard curve for calibration of the DNA, 1 × 10^6^ tumour cells of HT 29 cells were isolated using the same kit. The extracted DNA was resuspended in 200 *μ*l elution buffer (10 mM Tris, adjusted with 1 M HCl to pH 8.5) and a sequential dilution series was performed. The standard solutions that were previously prepared as a bulk preparation were diluted into murine DNA from untreated E- and P-selectin-deficient scid mice.

### Real-time PCR

HPLC-purified primers for the human carcinoembryonic antigen (CEA) as previously described (Katoh *et al*, 2004) and for the human consensus Alu sequence gene fragments consisting of the oligonucleotide sequences 5′-TGGCTCACGCCTGTAATCCCA-3′ (forward primer) and 5′-GCCACTACGCCCGGCTAATTT-3′ (reverse primer) were obtained from MWG-Biotech AG. PCR reactions were performed in 20 *μ*l volume capillaries (no. 1 909 339, Roche Diagnostics) containing 0.4 *μ*l of each primer (100 mM), 2 *μ*l of mastermix ( × 10 conc.), sterile PCR-grade distilled water added to a total volume of 18 and 2 *μ*l of sample DNA. The mastermix containing Taq DNA polymerase, reaction buffer ( × 10 conc.), SYBR Green I dye, dNTP mix (with dUTP instead of dTTP) and 10 mM MgCl_2_ was obtained from Roche Diagnostics (no. 003 752 186 001, LightCycler – FastStart DNA Master SYBR Green I, 100 *μ*l Reactions-Kit). Each capillary was sealed with a stopper and centrifuged at 3000 r.p.m. for 5 s.

RT–PCR was performed in the following steps:


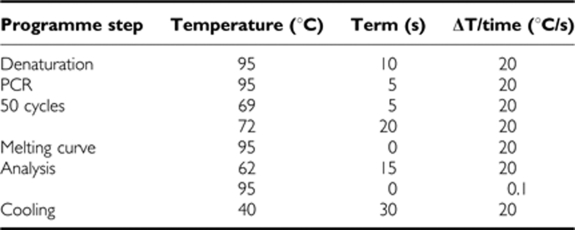


For quantification of the PCR products, a standard curve of human DNA fragments in a mouse background was used. The number of circulating human tumour cells was determined by interpolating the signal strength of the DNA template to an equivalent signal of the standard samples using the LightCycler software 4.05 (Roche).

### Laminar flow assay

Laminar flow was produced in IBIDI microslides VI (IBIDI; ibiTreat-pretreatment; width: 3.8 mm, height: 0.4 mm, volume of each capillary: 30 *μ*l) connected to a syringe pump (Model 100 Series, kdScientific, Holliston, MA, USA) and cell movement was observed with an inverted microscope (Zeiss, Axiovert 200) (Strell, 2007). HT 29 cells were suspended in cell culture medium (GIBCO-Invitrogen, Carlsbad, CA, USA, 40 ml, 130 000 c ml^–1^) and microslides were coated with recombinant human (rh) E- and P-selectin Fc-chimeras (R&D Systems) diluted in DPBS (GIBCO-Invitrogen) to 5and 50 *μ*g ml^–1^. A control capillary was incubated with RPMI-1640 and L-glutamine medium supplemented with 10% fetal calf serum to block nonspecific cell adhesion. To exclude tumour cell adhesion to the conjugated Fc-fragment, a microslide was coated with rh IgG1-Fc (R&D Systems) with the same concentrations as the fusion proteins. Physiological shear stress (Koutsiaris *et al*, 2007) was produced with a syringe pump (kdScientific, Model 100 Series), initially set to 0.55 dyn cm^–2^ for 5 min and subsequently raised to 5.0 and 10.0 dyn cm^–2^ for 2 min each. Cell movement was recorded and analysed with regard to the quality of movement (adhesion, rolling and tethering) and rolling velocity using a specially designed analysing program (CapImage 8.5, Dr Heinrich Zeintl, Heidelberg). Three independent experiments were conducted and analysed.

## Results

### Establishment of the E- and P- selectin-deficient scid mouse strain

After crossbreeding scid and E (−/−) and P (−/−) double selectin-deficient mice and selective backcrossing of defective mutant individuals, 27 F4 descendants and their F5 offspring were genotyped to be homozygous E- and P-selectin-deficient scid mice. Thus, 30 E- and P-selectin-deficient scid mice homozygous mutants lacking E- and P-selectin expression and 29 scid mice homozygous wild types for E- and P-selectin as controls were included in this study.

### E- and P-selectin deficiency delays growth of the primary HT 29 colon cancers and reduces the number of lung metastases

**Figure 1:**
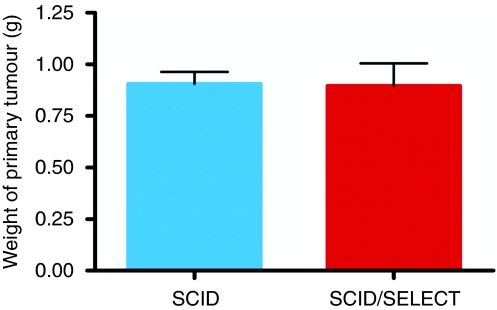
Weight of the xenografted HT 29 primary tumours. The weight of the primary tumours is identical and just below 1 g in both wild-type scid and E- and P-selectin-deficient scid mice.

**Figure 2:**
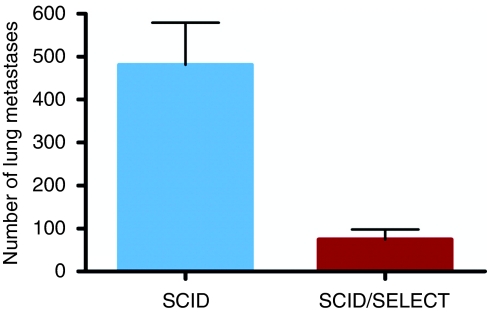
Number of spontaneous lung metastases in both mouse strains. Note that the number of metastases is reduced by 84% in E- and P-selectin-deficient scid mice in comparison with wild-type scid mice.

In general, mice were killed when the primary tumour ulcerated or the tumour weight exceeded 20% of the initial weight at the beginning of the experiment, except for three E- and P-selectin-deficient scid mice that were killed 53 days after cell transplantation showing no further tumour development. At the time of the killing, the weight of the solid primary tumours was identical between E- and P-selectin-deficient and wild-type scid mice: both were just below 1 g (Figure 1). Although solid tumours had grown in all scid mice within 2 weeks and grew rapidly in size thereafter, the tumour growth rate in E- and P-selectin-deficient mice was reduced. In contrast to the weight of the primary tumour, the mean value of the number of lung metastases was 481±98 in wild-type scid mice and 75±37 in E- and P-selectin-deficient scid mice, with the differences between the two groups being highly significant (*P*<0.0001; see Figure 2).

### Histology of the primary tumours and of the lung metastases

**Figure 3:**
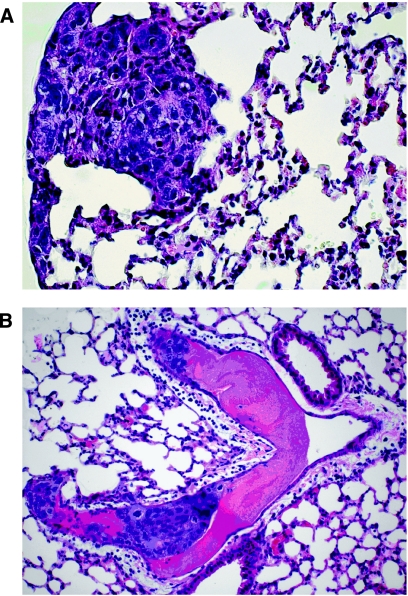
Histology of spontaneous HT 29 lung metastases. (**A**) Wild-type scid mouse (1 : 50) and (**B**) E- and P-selectin-deficient scid mouse (1 : 50), H&E stain. Although spontaneous metastasis had developed in the alveolar septum in the periphery of the lung in the wild-type scid mouse (**A**), the metastasis in the E- and P-selectin-deficient scid mouse had grown only within a branch of the pulmonary artery (**B**).

Differences in general morphology of the primary solid primary tumours and their vascularisation pattern between both mouse strains were not obvious. In general, primary tumours contained several signet ring cells and mitoses were frequently observed. The central parts of the tumours were often necrotic. However, not only the number but also the metastatic pattern was affected by E- and P-selectin deficiency. Although lung metastases grew in alveolar septae in wild-type scid mice, metastases were mainly found in pulmonary arteries and their tributaries in E- and P-selectin-deficient scid mice (Figure 3A and B). No intra-pulmonary artery metastases were present in the wild-type scid mice. In addition, in all E- and P-selectin-deficient scid mice, lung metastases were generally smaller than those in the scid mouse control group. In both mouse strains, no signet ring cells were present in the metastases.

### Lectin histochemistry

HPA labelling of HT 29 tumour cells grown in cell culture was highly positive (+++). Staining of primary HT 29 tumours was detectable in all mice, but varied considerably in both mouse strains; however, HPA-positive cells (++) were clearly present. In the lungs, only Clara cells of the bronchioli were positively stained with HPA, and no labelling of metastases was detected. In general, no variation of the staining pattern was observed between different animals.

### Selectin histochemistry

Labelling of paraffin wax embedded cell culture-grown HT 29 cells and in chamber slides grown HT 29 cells was strongly positive (+++) for both selectin fusion proteins. Tumour cells in primary tumours and in lung metastases were generally only labelled (++) when they were located adjacent to blood vessels. No difference of the staining pattern or intensity was observed in scid mice and E- and P-selectin-deficient scid mice. Positive controls for the E-selectin IgG fusion protein were carried out on human salivary gland tumour cells and human lung adenocarcinoma cells, the latter additionally for the P-selectin IgG fusion protein. Labelling was clearly detectable because nonspecific background effects were limited to necrotic parts of the tumours. Negative controls showed no staining.

### Double labelling with HPA and P-selectin IgG fusion protein

**Figure 4:**
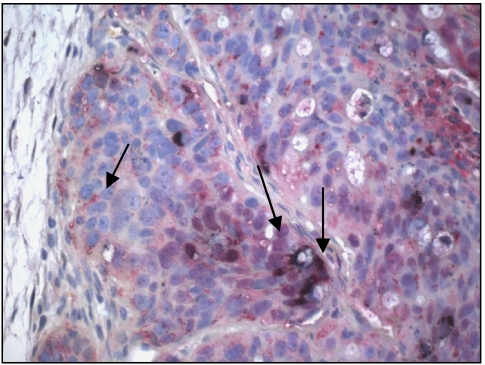
Double labelling of HT 29 cells with HPA and P-selectin IgG fusion protein. Double labelling of HT 29 cells of a primary tumour grown in a wild-type scid mouse with HPA (10 *μ*g ml^–1^; blue-black) and P-selectin IgG fusion protein (4 *μ*g ml^–1^; red). Note the unique red staining of some cells, whereas others show a unique dark blue staining. Only a few cells represent with a mixed colour, indicating that most of the cells express either HPA or P-selectin binding sites (1 : 100). A full-colour version of this figure is available at BJC online.

Double labelling of HT 29 cells in primary tumours of both mouse strains showed partly simultaneous labelling of the tumour cells with HPA and P-selectin IgG fusion protein. Some tumour cells were marked by a mixed colour, but most tumour cells were stained with a single colour (Figure 4).

### Staining with anti-human sLe^a^ and anti-human sLe^x^

**Figure 5:**
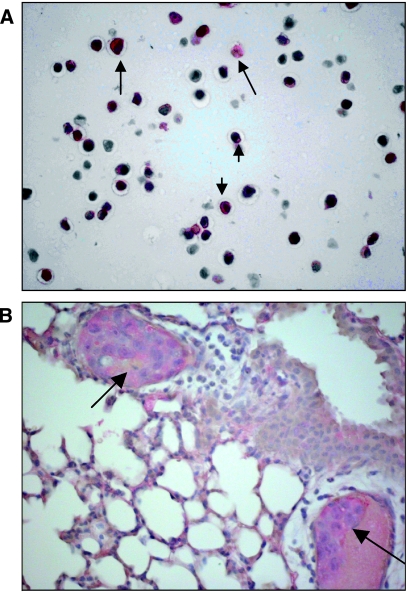
Labelling of HT 29 cells with anti-sLe^a^. (**A**) Approximately 30% of the HT 29 tumour cells grown in cell culture are intensively labelled with an anti-sialyl Lewis a antibody (1 : 100). (**B**) Intra-arterial metastases (arrows) in an E- and P-selectin-deficient scid mouse are faintly labelled by anti-sLe^a^.

HT 29 tumour cells grown in cell culture were clearly labelled by the antibody raised against the human selectin ligand sLe^a^ (clone 121 SLE) (+++), whereas primary tumours and lung metastases in both mouse strains showed a less intensive staining (+) (see Figure 5). When HT 29 tumour cells grown in cell culture were preincubated with the antibody against sLe^a^, the subsequent staining with the E- or P-selectin IgG fusion protein was not blocked.

**Figure 6:**
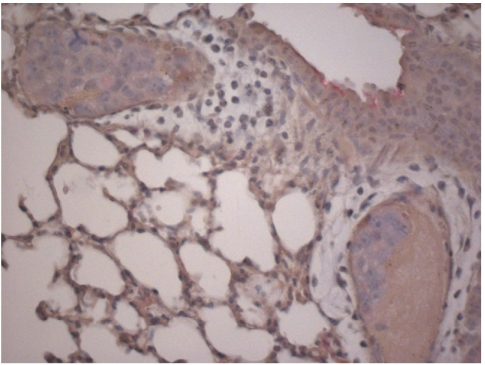
Labelling of HT 29 cells with anti-sLe^x^. Parallel section of the metastases shown in Figure 5b labelled with an anti-sialyl Lewis x antibody (1 : 100). In comparison with the anti-sLe^a^ staining, hardly any anti-sLe^x^ is present.

In contrast, no staining of the HT 29 tumours with an antibody raised against the human selectin ligand sLe^x^ (CD15s) was observed (Figure 6). Primary tumours of the human breast cancer cell line, DU4475, used as a positive control were clearly labelled (++). HT 29 tumour cells in cell culture also showed only a weak labelling similar to the staining result of metastatic cells in the lung.

### Tumour cells in the bloodstream

The mean value of the number of circulating HT 29 tumour cells was 1364±418/200 *μ*l mouse blood in wild-type scid mice (*n*=23) in comparison with 2282±657/200 *μ*l mouse blood in E- and P-selectin-deficient scid mice (*n*=21) using the CEA sequences, the difference between both groups being highly significant (*P*<0.0001). Similar results were obtained using the Alu sequences, which, however, showed a higher background level (data not shown).

### Cell flow assay

#### Rh E-selectin–FC-chimera coated microslides

In general, HT 29 cells showed both firm adhesion and rolling movements on an rh E-selectin–Fc-chimera coated surface with both selectin concentrations (5 and 50 *μ*g ml^–1^), whereas there was no adhesion or rolling in the control capillaries either incubated with cell culture medium or coated with the Fc-fragment.

**Figure 7:**
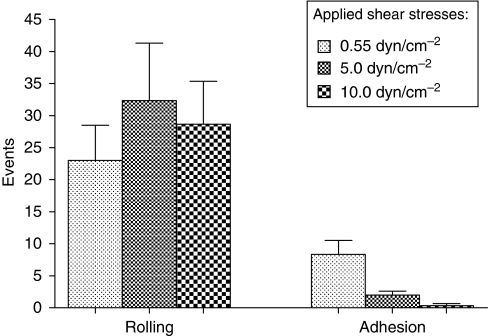
Flow assay of HT 29 cells on E- and P-selectin-coated slides. Number of adhesive and rolling HT 29 cells at different shear stresses on rh E-selectin–Fc-chimera coated microslides with a concentration of 5 *μ*g ml^–1^.

With an rh E-selectin fusion protein concentration of 5 *μ*g ml^–1^, the mean number of rolling HT 29 cells at 0.55 dyn cm^–2^ per time unit and field of view (0.8 × 0.6 mm) was 23.0 (Figure 7). At 5.0 dyn cm^–2^, the number increased to 32.33 rolling cells and was 28.67 at 10.0 dyn cm^–2^. At 0.55 dyn cm^2^, the mean number of adhering cells was 8.33, which dropped to 0.33 cells at 10.0 dyn cm^–2^.

With an rh E-selectin fusion protein concentration of 50 *μ*g ml^–1^, the mean number of rolling tumour cells was 10 at 0.55 dyn cm^–2^ and increased to 12.7 at 10.0 dyn cm^–2^. The number of firmly adhering cells dropped from 17 at 0.55 dyn cm^–2^ to 10.3 at 10.0 dyn cm^–2^.

The mean cell rolling velocity at 0.55 dyn cm^–2^ was 1.623 *μ*m s^–1^. At 5.0 dyn cm^–2^, it increased to 2.443 *μ*m s^–1^ and dropped to 2.244 *μ*m s^–1^ at 10 dyn cm^–2^.

#### Rh P-selectin–FC-chimera coated microslides

With an rh P-selectin fusion protein concentration of 5 *μ*g ml^–1^, no events could be observed at any shear stress. With a concentration of 50 *μ*g ml^–1^ and a shear stress of 0.55 dyn cm^–2^, no firm cell adhesion or steady cell rolling could be observed except for loose tethering movements, alternating cell adhesion and breaking away from the coated ground. The mean number of tethering events at 0.55 dyn cm^–2^ was 112.7. At shear stresses of 5.0 and 10.0 dyn cm^–2^, no cell tethering could be observed.

## Discussion

The major aim of this study was to determine whether E- and P-selectins have a functional role in metastasis using a clinically relevant xenograft model of spontaneous metastasis formation. Indeed, the number of lung metastases after subcutaneous implantation of the human HT 29 colon cancer cells into E- and P-selectin-deficient scid mice was very significantly reduced by 84% when compared with wild-type scid mice (Figure 2). Although our model is the first to show the importance of E- and P-selectins in spontaneous metastasis formation, in which the metastatic tumour cell has to undergo the complete metastatic cascade, earlier models focused on the prevention of dissemination, that is, the lodging of intravenously injected tumour cells in P-selectin-deficient mice (Kim *et al*, 1998) or by blocking E-selectin using cimetidine (Kobayashi *et al*, 2000).

Selectins are known to mediate adhesion of leukocytes to endothelial cells and to sustain their transmigration through the vascular endothelium into the stroma of the target organ. Thus, it is easily imaginable that selectin–ligand interactions between endothelia and tumour cells use the same molecular mechanisms as leukocytes do. This hypothesis is underscored by our finding that a higher number of circulating tumour cells was observed in the bloodstream of E- and P-selectin-deficient scid mice compared with wild-type scid mice, indicating that E- and P-selectin deficiency affected tumour cells extravasation as in E- and P-selectin-deficient mice, in which an extreme leukocytosis was observed, indicating that the leukocytes were similarly unable to leave the circulation (Frenette *et al*, 1996). In addition, our HT29 tumour cells behaved in the same manner with rolling and tethering just as leucocytes do in the flow assay. The importance of the selectins for extravasation was also evident when looking at the metastatic growth pattern in E- and P-selectin-deficient scid mice. Although metastases had engrafted in the connective tissue of the alveolar septae in wild-type scid mice, metastases in the E- and P-selectin-deficient scid mice had only grown in the pulmonary arteries and their tributaries (Figure 3A and B) – a rare occurrence in humans – indicating that the metastasising tumour cells were unable to cross the endothelial barrier. In contrast to the considerable reduction in the number of spontaneous lung metastases, the growth of the primary tumours was only marginally affected by E- and P-selectin deficiency (Figure 1). Taken together, our findings indicate an important functional role of E- and P-selectins in the adhesion and extravasation process of metastatic tumour cells into the stroma of their target organs.

Thus, the question arises of whether E- and P-selectin work synergistically or which of the two selectins is required for the adhesive and trafficking effects in question. Although the colon cancer cell lines LS 180, T 84 and COLO 205 have been shown to bind to recombinant E-, P- and L-selectins, the colon cancer cell line COLO 320 bound to P- and L-selectins, but not to E-selectin. In contrast, HT 29 cells have been found to bind to E-selectin only, but not to P- or- L-selectins, whereas colon cancer Caco-2 cells showed little or no interaction with any of the three selectins (Mannori *et al*, 1995). The latter result is partly in agreement with our own analysis; however, we could show the presence of both E- and P-selectin binding sites on HT 29 cells grown *in vitro*. Furthermore, adhesion of HT 29 cells to human umbilical venous endothelial cells (HUVECs) was shown to be E-selectin dependent, whereas probably a complex of adhesion molecules was responsible for enhanced HT 29 cell adhesion to monolayers of microvascular endothelial cells of the lungs (HMVECs-L) (ten Kate *et al*, 2004; for HUVECs also Lafferière *et al*, 2004). Taken together, similar to leukocyte diapedesis, several adhesive factors seem to be required for metastasis formation. Therefore, we thought it sensible to start our investigation with the E- and P-selectin double knockout mice. Having found that the metastatic rate is drastically reduced by E- and P-selectin deficiency, further analysis are now under way to elaborate the role of each selectin individually.

To discriminate between the individual roles of E- and P-selectin in the course of extravasation, an *in vitro* cell flow assay was performed simulating the physiologic shear stresses in post-capillary venules. This experiment revealed that HT 29 cells firmly adhered and rolled on E-selectin fusion protein-coated capillaries with a low concentration of 5 *μ*g ml^–1^ and a shear stress of up to 10 dyn cm^–2^ but did not so on P-selectin fusion protein coated microslides even with a concentration 10 times higher than that used for E-selectin. With P-selectin fusion protein-coated capillaries, only loose tethering could be observed, indicating that the binding strength of the HT29 tumour cells to E-selectin is much higher than to P-selectin. This result agrees with the abovementioned finding that HT 29 cells bind to E-selectin only, but not to P- or L-selectins (Mannori *et al*, 1995).

Tumour cell-endothelia interactions do not depend only on the presence of the particular selectin on the luminal surface of endothelial cells but also on the presence of selectin ligands on the tumour cell surface. HT 29 cells grown in cell culture were clearly labelled by anti-sLe^a^, whereas primary tumours and lung metastases showed a less intensive staining (Figure 5). In contrast, one of the major classical selectin-binding structures, namely sLe^x^, was not present on the *in vivo* grown HT 29 tumours in both mouse strains, whereas it was only weakly present on the tumour cells of HT 29 cells grown in cell culture and in the metastatic deposits (Figure 6). These findings suggest that the major selectin ligand present on HT 29 cells is sLe^a^. However, preincubation with anti-sLe^a^ did not block following staining with E- or P-selectin IgG fusion protein, thus indicating the presence of diverse selectin-binding structures on HT 29 cells. sLe^x^ is a tetrasaccharide built on galactose, *N*-acetylglucosamine and fucose (Brooks *et al*, 2002). As HPA also recognises *N*-acetylglucosamine (Hammarström *et al*, 1977), and the HPA-binding structure has been identified as a mono-sialylated tetrasaccharide (Dwek *et al*, 2001), it is attractive to speculate that the glycotope recognised by HPA is similar but not identical to an sLe^x^ glycotope, as the binding patterns of HPA and anti-sLe^x^ antibodies differed strongly in this investigation. This similarity can also be extended to sLe^a^ and HPA: only some HT 29 cells showed a mixed colour of HPA and P-selectin fusion protein staining, whereas most of them bound either HPA or P-selectin fusion protein (Figure 4), indicating that the glycotopes recognised by the selectins and HPA are different and that most – but not all – of the tumour cells express either selectin or HPA-binding sites. The hypothesis of the existence of diverse selectin ligands was furthermore accompanied by another interesting observation: none of the expressions of these binding sites was static. Using the E- and P-selectin fusion proteins, an intensive labelling of all HT 29 cells was noted when they had been grown *in vitro*, whereas only labelling of some HT 29 tumour cells in primary tumours grown in both strains of mice was noted, and in particular when the tumour cells had grown adjacent to blood vessels. A similar phenomenon was found by labelling with HPA and anti-sLe^a^ and no difference in the binding pattern or intensity was detected between both strains of mice. This observation leads to the idea that the expression of selectin ligands of the tumour cells can vary and that their selectin-binding pattern is probably influenced by the different environment of the tumour cells, as already shown by Koike *et al* (2004), who showed that oxygenation influences selectin-binding site expression. This difference in expression pattern is explained by the observation that transformational effects, such as growth to high cell densities, 2D *vs* 3D growth or hypoxia, lead to an alteration of carbohydrate patterns between tumour cells grown *in vivo* and *in vitro* (Warren *et al*, 1978). Hence, binding pattern and metastatic potential of tumour cells as indicated by selectin and HPA-binding site expression are not static features, but may be subjected to a dynamic process as observed during the epithelial mesenchymal transition (EMT) of tumour cells. During the EMT, the tumour cells lose their contact with their neighbouring epithelial cells and basal lamina, respectively, and acquire a migratory (=mesenchymal) phenotype (Berx *et al*, 2007). Taking this thought further, it would also indicate that the ability to metastasise is at least in part subject to regulatory mechanisms.

In summary, in this study the considerable reduction in the number of spontaneous lung metastases of the human colon cancer cell line HT 29 in E- and P-selectin-deficient scid mice could be quantified *in vivo*. This finding highlights the importance of the selectins for metastasis formation. Our selectin-binding site studies furthermore indicate that the glycotope expression, and thus the metastatic potential of tumour cells, is subject to a dynamic process similar to the other phenotypical changes observed during the EMT of tumour cells.

## References

[bib1] Altevogt P, Fogel M, Cheingsong-Popov R, Dennis J, Robinson P, Schirrmacher V (1983) Different patterns of lectin binding and cell surface sialylation detected on related high- and low-metastic tumour lines. Cancer Res 43: 5138–5144 6616451

[bib2] Berx G, Raspé E, Christofori G, Thiery JP, Sleeman JP (2007) Pre-EMTing metastasis? Recapitulation of morphogenetic processes in cancer. Clin Exp Metastasis 24: 587–597 17978854 10.1007/s10585-007-9114-6

[bib3] Bosma GC, Custer RP, Bosma MJ (1983) A severe combined immunodeficiency mutation in the mouse. Nature 301: 527–530 6823332 10.1038/301527a0

[bib4] Brittan M, Hunt T, Jefferey R, Poulsom R, Forbes SJ, Hodivala-Dilke K, Goldmann J, Alison MR, Wright NA (2002) Bone marrow derivation of pericryptal myofibroblasts in the mouse and human small intestine and colon. Gut 50: 752–757 12010874 10.1136/gut.50.6.752 PMC1773238

[bib5] Brooks SA (2000) The involvement of Helix pomatia lectin (HPA) binding N-acetylgalactosamine glycans in cancer progression. Histol Histopathol 15: 143–158 10668205 10.14670/HH-15.143

[bib6] Brooks SA, Dwek MV, Schumacher U (2002) Functional and Molecular Glycobiology. Bios Scientific Publishers: Oxford, p 214

[bib7] Brooks SA, Lymboura M, Schumacher U, Leathem AJ (1996) Histochemistry to detect *Helix pomatia* lectin binding in breast cancer: methodology makes a difference. J Histochem Cytochem 44: 519–524 8627008 10.1177/44.5.8627008

[bib8] Brown KL, Stewart K, Bruce ME, Fraser H (1997) Severely combined immunodeficient (SCID) mice resist infection with bovine spongiform encephalopathy. J Gen Virol 78: 2707–2710 9349494 10.1099/0022-1317-78-10-2707

[bib9] Chambers AF, Groom AC, MacDonald IC (2002) Dissemination and growth of cancer cells in metastatic sites. Nat Rev Cancer 2: 563–572 12154349 10.1038/nrc865

[bib10] Dwek MV, Ross HA, Streets AJ, Brooks SA, Adam E, Titcomb A, Woodside JV, Schumacher U, Leathem AJ (2001) Helix pomatia agglutinin lectin-binding oligosaccharides of aggressive breast cancer. Int J Cancer 95: 79–85 11241316 10.1002/1097-0215(20010320)95:2<79::aid-ijc1014>3.0.co;2-e

[bib11] Frenette PS, Mayadas TN, Rayburn H, Hynes RO, Wagner DD (1996) Susceptibility to infection and altered hematopoiesis in mice deficient in both P- and E-selectins. Cell 84: 563–574 8598043 10.1016/s0092-8674(00)81032-6

[bib12] Hammarström S, Murphy LA, Goldstein IJ, Etzler ME (1977) Carbohydrate binding specificity of four N-acetyl-D-galactosamine-‘specific’ lectins: Helix pomatia A hemagglutinin, soy bean agglutinin, lima bean lectin, and Dolichos biflorus lectin. Biochemistry 16: 2750–2755 560855 10.1021/bi00631a025

[bib13] Jojovic M, Schumacher U (2000) Quantitative assessment of spontaneous lung metastases of human HT-29 colon cancer cells transplanted into scid mice. Cancer Lett 153: 151–156 10773406 10.1016/s0304-3835(99)00443-7

[bib14] Katoh M, Neumaier M, Nezam R, Izbicki JR, Schumacher U (2004) Correlation of circulating tumor cells with tumor size and metastatic load in a spontaneous lung metastasis model. Anticancer Res 24: 1421–1425 15274304

[bib15] Kim YJ, Borsig L, Varki NM, Varki A (1998) P-selectin deficiency attenuates tumor growth and metastasis. Proc Natl Acad Sci USA 95: 9325–9330 9689079 10.1073/pnas.95.16.9325 PMC21337

[bib16] Kobayashi K, Matsumoto S, Morishima T, Kawabe T, Okamoto T (2000) Cimetidine inhibits cancer cell adhesion to endothelial cells and prevents metastasis by blocking E-selectin expression. Cancer Res 60: 3978–3984 10919677

[bib17] Koike T, Kimura N, Miyazaki K, Yabuta T, Kumamoto K, Takenoshita S, Chen J, Kobayashi M, Hosokawa M, Tanijuki A, Kojima T, Ishida N, Kawakita M, Yamamoto H, Takematsu H, Suzuki A, Kozutsumi Y, Kanangi R (2004) Hypoxia induces adhesion molecules on cancer cells: a missing link between Warburg effect and induction of selectin-ligand carbohydrates. Proc Natl Acad Sci USA 101: 8132–8137 15141079 10.1073/pnas.0402088101 PMC419569

[bib18] Konno A, Hoshino Y, Terashima S, Motoki R, Kawaguchi T (2002) Carbohydrate expression profile of colorectal cancer cells is relevant to metastatic pattern and prognosis. Clin Exp Metastasis 19: 61–70 11918084 10.1023/a:1013879702702

[bib19] Koutsiaris AG, Tachmitzi SV, Batis N, Kotoula MG, Karabatsas CH, Tsironi E, Chatzoulis DZ (2007) Volume flow and wall shear stress quantification in the human conjunctival capillaries and post-capillary venules *in vivo*. Biorheology 44: 375–386 18401076

[bib20] Lafferière J, Houle F, Huot J (2004) Adhesion of HT-29 colon carcinoma cells to endothelial cells requires sequential events involving E-selectin and integrin *β*4. Clin Exp Metastasis 21: 257–264 15387376 10.1023/b:clin.0000037708.09420.9a

[bib21] Mannori G, Crottet P, Cecconi O, Hanasaki K, Aruffo A, Nelson RM, Varki A, Bevilacqua MP (1995) Differential colon cancer cell adhesion to E-, P-, and L-selectin: role of mucin-type glycoproteins. Cancer Res 55: 4425–4431 7545541

[bib22] Mitchell BS, Schumacher U (1999) The use of the lectin *Helix pomatia* agglutinin (HPA) as a prognostic indicator and as a tool in cancer research. Histol Histopathol 14: 217–226 9987666 10.14670/HH-14.217

[bib23] Mitchell BS, Vernon K, Schumacher U (1995) Ultrastructural localization of Helix pomatia agglutinin (HPA)-binding sites in human breast cancer cell lines and characterization of HPA-binding glycoproteins by western blotting. Ultrastruct Pathol 19: 51–59 7770962 10.3109/01913129509014603

[bib24] Napier SL, Healy ZR, Schnaar RL, Konstantopoulos K (2007) Selectin ligand expression regulates the initial vascular interactions of colon carcinoma cells: the roles of CD44v and alternative sialofucosylated selectin ligands. J Biol Chem 282: 3433–3441 17135256 10.1074/jbc.M607219200

[bib25] Poston GJ, Figueras J, Giuliante F, Nuzzo G, Sobrero AF, Gigot JF, Nordlinger B, Adam R, Gruenberger T, Choti MA, Bilchik AJ, Van Cutsem EJ, Chiang JM, D’Angelica MI (2008) Urgent need for a new staging system in advanced colorectal cancer. J Clin Oncol 26: 4828–4833 18711170 10.1200/JCO.2008.17.6453

[bib26] Rajan S, Ye J, Bai S, Huang F, Guo YL (2008) NF-kappaB, but not p38 MAP Kinase, is required for TNF-alpha-induced expression of cell adhesion molecules in endothelial cells. J Cell Biochem 105: 477–486 18613029 10.1002/jcb.21845 PMC4422387

[bib27] Resto VA, Burdick MM, Dagia NM, McCammon SD, Fennewald SM, Sackstein R (2008) L-selectin-mediated lymphocyte-cancer cell interactions under low fluid shear stress conditions. J Biol Chem 283: 15816–15824 18385135 10.1074/jbc.M708899200 PMC2414279

[bib28] Schumacher U, Adam E (1997) Lectin histochemical HPA-binding pattern of human breast and colon cancers is associated with metastases formation in severe combined immunodeficient mice. Histochem J 29: 677–684 9413741 10.1023/a:1026404832394

[bib29] Schumacher U, Adam E, Flavell DJ, Boehm D, Brooks SA, Leathem AJ (1994) Glycosylation patterns of the human colon cancer cell line HT-29 detected by Helix pomatia agglutinin and other lectins in culture, in primary tumours and in metastases in SCID mice. Clin Exp Metastasis 12: 398–404 7923992 10.1007/BF01755883

[bib30] Sperandio M (2006) Selectins and glycosyltransferases in leukocyte rolling *in vivo*. FEBS J 273: 4377–4389 16956372 10.1111/j.1742-4658.2006.05437.x

[bib31] Strell C, Lang K, Niggemann B, Zaenker KS, Entschladen F (2007) Surface molecules regulating rolling and adhesion to endothelium of neutrophil granulocytes and MDA-MB-468 breast carcinoma cells and their interaction. Cell Mol Life Sci 64: 3306–3316 17994288 10.1007/s00018-007-7402-6 PMC11136373

[bib32] ten Kate M, Hofland LJ, van Grevenstein WM, van Koetsveld PV, Jeekel J, van Eijck CH (2004) Influence of proinflammatory cytokines on the adhesion of human colon carcina cells to lung microvascular endothelium. Int J Cancer 112: 943–950 15386356 10.1002/ijc.20506

[bib33] Thomas SN, Schnaar RL, Konstantopoulos K (2009) Podocalyxin-like protein is an E-/L-selectin ligand on colon carcinoma cells: comparative biochemical properties of selectin ligands in host and tumor cells. Am J Physiol Cell Physiol 296: C505–C513 19118161 10.1152/ajpcell.00472.2008 PMC2660269

[bib34] Thomas SN, Zhu F, Schnaar RL, Alves CS, Konstantopolous K (2008) Carcinoembryonic antigen and CD44 variant isoforms cooperate to mediate colon carcinoma cell adhesion to E- and L-selectin in shear flow. J Biol Chem 283: 15647–15655 18375392 10.1074/jbc.M800543200 PMC2414264

[bib35] Tremblay PL, Huot J, Auger FA (2008) Mechanisms by which E-selectin regulates diapedesis of colon cancer cells under flow conditions. Cancer Res 68: 5167–5176 18593916 10.1158/0008-5472.CAN-08-1229

[bib36] Valentiner U, Hall DMS, Brooks SA, Schumacher U (2005) Transplantation of human breast cancer cell lines into scid mice: HPA binding and metastasis formation of human breast cancer cell lines transplanted into severe combined immunodeficient (scid) mice. Cancer Lett 219: 233–242 15723724 10.1016/j.canlet.2004.07.046

[bib37] Warren L, Clayton AB, Tuszynski GP (1978) Glycopeptide changes and malignant transformation – a possible role for carbohydrate in malignant behaviour. Biochemica et Biophysica Acta 516: 97–127 10.1016/0304-419x(78)90005-7 361084

